# Backlash against gender stereotype-violating preschool children

**DOI:** 10.1371/journal.pone.0195503

**Published:** 2018-04-09

**Authors:** Jessica Sullivan, Corinne Moss-Racusin, Michael Lopez, Katherine Williams

**Affiliations:** 1 Department of Psychology, Skidmore College, Saratoga Springs, United States of America; 2 Department of Mathematics, Skidmore College, Saratoga Springs, United States of America; University of Birmingham, UNITED KINGDOM

## Abstract

While there is substantial evidence that adults who violate gender stereotypes often face backlash (i.e. social and economic penalties), less is known about the nature of gender stereotypes for young children, and the penalties that children may face for violating them. We conducted three experiments, with over 2000 adults from the US, to better understand the content and consequences of adults’ gender stereotypes for young children. In Experiment 1, we tested which characteristics adults (*N* = 635) believed to be descriptive (i.e. typical), prescriptive (i.e. required), and proscriptive (i.e. forbidden) for preschool-aged boys and girls. Using the characteristics that were rated in Experiment 1, we then constructed vignettes that were either ‘masculine’ or ‘feminine’, and manipulated whether the vignettes were said to describe a boy or a girl. Experiment 2 (*N* = 697) revealed that adults rated stereotype-violating children as less likeable than their stereotype-conforming peers, and that this difference was more robust for boys than girls. Experiment 3 (*N* = 731) was a direct replication of Experiment 2, and revealed converging evidence of backlash against stereotype-violating children. In sum, our results suggest that even young children encounter backlash from adults for stereotype violations, and that these effects may be strongest for boys.

## Introduction

Psychologists Sandra and Daryl Bem famously sought to raise their children without the constraints of gender stereotypes [1]. Specifically, the Bems encouraged both Jeremy and Emily to wear whatever clothes and accessories they loved, play with whatever toys were compelling to them, and behave in whatever ways felt natural, regardless of whether these things were typically ascribed to girls or boys. Although this “gender aschematic” parenting was in some ways successful, the Bems also encountered roadblocks (in the form of negative reactions from others) that are emblematic of the strong role of gendered expectations in shaping the behavior of even very young children [1]. For example, in one much-shared anecdote, when Jeremy decided to wear pink barrettes to preschool, a male classmate taunted him repeatedly, claiming that “Jeremy must be a girl, not a boy, because ‘only girls wear barrettes’” [1]. This case study suggests that even very purposeful attempts to mitigate the early impact of gendered expectations often encounter pushback from the broader culture.

Indeed, within the first years of life, children develop increasingly rigid beliefs about the behaviors, preferences, and traits associated with particular genders (see [2–16]). The internalization of these stereotypes can profoundly shape an individual’s goals and actions [17]. More generally, we know that children’s books convey strong messages about gender stereotypical behavior [18], that parent-child play and conversation are impacted by both gender and gender stereotypes [19–22], and that children often receive positive feedback when they conform to gender stereotypes [23]. One possible explanation for children’s development of strong gender stereotypes early in life is that structures in adult society provide strong cues to gender stereotypical behaviors [23–24]. However, this raises an additional question: what is the nature of adults’ gender stereotypes of children, and what are the consequences of these stereotypes? In the present study, we investigated the nature and consequences of adults’ gender stereotypes about young children.

There is strong evidence that adults penalize other adults who violate gender stereotypes [25], raising the possibility that adults might also penalize children who violate gender stereotypes. In order to understand how such penalizations function, it is important to first clarify the content and nature of gender stereotypes about adults. In general, women are stereotyped as being communal (e.g., supportive, nurturing), while men are stereotyped as being agentic (e.g., independent, able to lead [26]). However, gender stereotypes take three distinct forms. Descriptive stereotypes describe how individuals of a particular gender are *typically* perceived. When an individual violates a descriptive gender stereotype, they are rarely penalized for doing so [27]; instead, individuals may treat a descriptive stereotype-violating individual as an exception to the rule (i.e. individuation), or may create a new subtype of their existing stereotype to accommodate these new “data” (i.e. sub-typing; [28–29]).

However, stereotypes can also be prescriptive (what an individual *should* be like/do; e.g., [30]), as well as proscriptive (what an individual *should not* be like/do; [31]). In contrast to those who violate descriptive stereotypes, adults who violate prescriptive and proscriptive stereotypes often encounter backlash (i.e. social and economic penalties [32–35]). Backlash manifests as prejudice (i.e. social penalties, reflecting the fact that stereotype-violators are liked less than stereotype-conformers), and sometimes also discrimination (i.e. economic penalties, as when stereotype-violators are less likely to be hired than stereotype-conformers; [36–38]). Typically, although stereotype-violating individuals are acknowledged as equally competent relative to stereotype-conforming counterparts when their credentials are made clear, they still suffer social (and sometimes economic) penalties [25]. Indeed, while competence penalties are rare and economic penalties are not universal, social penalties (e.g., being rated as less likeable) appear to be a consistent reaction to stereotype-violators [39]). Thus, dislike is the primary indicator of backlash.

There is evidence of backlash against gender stereotype-violating adults across a host of contexts. For example, women encounter backlash when they violate gender stereotypes by seeking political office [40], expressing anger [41], or vying for a managerial role [39]. Although the majority of existing work has focused on backlash against stereotype-violating women, recent evidence suggests that stereotype-violating men encounter backlash as well. For example, men encounter backlash when they violate gender stereotypes by working in early elementary education [42], requesting a family leave from work [43], and behaving modestly on a job interview [44]. Indeed, stereotype-violating men may actually be more susceptible to backlash than women [39]. Specifically, while women typically experience backlash only when displaying female-proscriptive traits (but not when they fail to display female-prescriptive traits), men experience backlash when they display male-proscriptive traits *and* when they fail to display male-prescriptive traits [25].

While we know a substantial amount about the content and consequences of gender stereotypes for adults, there is little experimental evidence of the content or consequences of adults’ gender stereotypes about children. There is some experimental evidence that adults treat infants and children who are labeled as girls differently from those who are labeled as boys ([45–48], but see [49–50]). For example, when shown a video of an infant reacting negatively to a stimulus, one study found that participants were more likely to label the infant’s reaction as anger if they believed the infant to be a boy, and fear if they believed it to be a girl ([45]; but see [49,51]).

There is also some limited experimental evidence that adults treat gender stereotype-violating children differently from stereotype-conforming children, and that this effect may be largest for stereotype-violating boys [52–54]. For example, boys labeled as “sissies,” boys described as feminine, and boys described as playing with girls’ toys were rated as less acceptable than stereotype-violating girls [53, 55]. Interestingly, adults also appear to be more likely to assume that stereotype-violating children are gay/lesbian, relative to stereotype-conforming individuals, and this effect appears largest for stereotype-violating boys [55–56]. Further, adults respond more negatively to children when they were engaged in “cross-sex-preferred” activities [20, 53–54].

Despite the presence of several studies (described above) exploring the role of gender in shaping perceptions of children, there has been no systematic study of the traits that adults consider to be descriptive, prescriptive, and proscriptive for young boys and girls. More generally, while we have anecdotal evidence [1] and limited experimental evidence [20, 54, 55] for backlash against gender stereotype-violating children, there has been no systematic investigation of gender backlash against children by adults. Specifically, to our knowledge, none of the work investigating the consequences of gender-stereotype-violation in children has tested for evidence of backlash using the gold-standard methods typical in studies of backlash against gender stereotype-violating adults: by systematically manipulating, between-subjects, both (a) the nature of a target’s gendered behavior and (b) the target’s stated sex. Instead, parents’ spontaneous interactions with their own children were coded [20], or college students were asked explicitly e.g., about the acceptability of being a “sissy” as a child, sometimes as part of a larger survey on gender stereotypes [54–55]. While these studies provide some of the first insights into adults’ judgments about gender-stereotype violating behavior in children, the small sample sizes and restricted participant pool limit the conclusions that we can (and should) draw. Perhaps more importantly, demand characteristics and social desirability constraints may have impacted performance on these previous tasks, and the lack of a true experimental design limits our ability to draw conclusions about backlash against children.

Given the current state of the literature, it is not immediately clear whether the content of gender stereotypes for children is similar to those for adults. That is, we do not yet know whether descriptive, prescriptive, and proscriptive stereotypes for children map onto those for adults, or whether adults might have different types of gendered expectations for children than for other adults. Further, it is not immediately apparent whether we should expect backlash against stereotype-violating children at all. On the one hand, because we know that adults experience gender backlash, it might be reasonable to assume that children will as well. On the other hand—at least in Western society—children are often thought of as needing protection, and their actions, even when failing to conform to cultural norms, frequently go unpenalized [57]. In addition, gender backlash is often most robust in cases where stereotype-violations disrupt existing power hierarchies (e.g., when women strive for power [38]). Because children’s stereotype-violations are unlikely to be viewed as posing a direct threat to the existing gender power hierarchy, children may be less likely than adults to encounter backlash for disconfirming gender stereotypes.

Gaining a better understanding of the impact of gender stereotypes on young children is important for at least three reasons. First, there is correlational evidence that gender non-conforming and non-binary adolescents are often bullied, and that this bullying increases mental distress and suicidality [58–59]; gaining insight into the early obstacles that stereotype-violating children may face is therefore of critical importance. Second, backlash against children may contribute to a vicious cycle, whereby those most likely to challenge existing stereotypes (i.e. stereotype-violators) are unwilling to do so, for fear of experiencing social [60]. As a result of gender backlash, the underlying gender stereotypes may then be reinforced, and applied to children as well as adults. Third, as described earlier, gender stereotypes are acquired early by children—understanding the ways in which adults enforce gender stereotypes may give insight into the mechanisms supporting the development of children’s gender cognition.

### Present study

The present research contains three preregistered experiments designed to systematically test the content of descriptive, prescriptive, and proscriptive gender stereotypes for preschool-aged children (Exp. 1), and to determine whether preschoolers who violate gender stereotypes encounter backlash from adults (Exps. 2 and 3). To do this, we conducted three online studies, recruiting a total sample of over 2,000 English speakers located in the US. In Experiment 1, we modified existing methods from the adult literature [38] to test which traits and behaviors adults consider to be descriptive, prescriptive, and proscriptive for three-year-old boys and girls. The results of Experiment 1 not only allowed us to provide novel insight into the content of gender stereotypes for young boys and girls, but also to construct rigorously controlled stimuli to test for evidence of adults’ gender backlash against. Specifically, in Experiments 2 and 3, we explored whether stereotype-violating children encounter backlash by presenting adults with three-year-old “applicants” for preschool—these applicants were labeled as either boys or girls, and were also described in short vignettes. These vignettes were either 'masculine' (containing male-descriptive, male-prescriptive, and female-proscriptive characteristics) or 'feminine' (containing female-descriptive, female-prescriptive, and male-proscriptive characteristics). We then measured a host of indicators of gender backlash modified from the existing adult literature including the primary measure of backlash (i.e., the likeability of the applicant) and other, potentially secondary measures (e.g., the applicant’s desirability as a student; [25]), as well as novel indicators of backlash against children developed for the purposes of the current research.

## Experiment 1

### Method

#### Participants

Data were collected from 635 adults 18 years or older. All participants were recruited from Amazon Mechanical Turk. Any person who had completed between 100 and 10,000 prior HITs (Human Intelligence Tasks on Amazon Mechanical Turk), had at least a 97% approval rating for prior HITs, and was a fluent English speaker from the US was eligible to participate. Participants received $1 compensation for their participation in the study.

#### Material and procedure

Participants completed the task independently on their own computer using the survey program Qualtrics; we used Turk Prime to manage participant recruitment [61]. The study took an average of 9 minutes to complete, and included 96 questions and two attention checks, displayed in random order across four pages.

Upon consenting, participants were randomly assigned to one of four conditions; half of the participants were asked to rate how **typical** particular characteristics were (reflecting descriptive judgements), and half were asked to rate how **desirable** particular characteristics were (reflecting prescriptive and proscriptive judgements). Additionally, half of the participants were asked to rate the characteristics for 3-year-olds **girls**, and half were asked to rate characteristics for 3-year-old **boys**. Participants then saw the following instructions: “In this study, we are interested in your beliefs about 3-year-old [girls/boys]. You will see some descriptions about [girls/boys], and you will be asked to make judgments about them. Please think about what is [desirable/typical] for 3-year-old [girls/boys].”

Participants then provided ratings for each of the characteristics. To develop the list of characteristics to be rated, we first began with the adult literature [38], and selected items that could be reasonably extended to children; next, we added additional characteristics that were likely to be specific to children, as informed by the previous literature [62], along with other characteristics that have been observed to be gendered (full study materials available at: https://osf.io/7rtca/). Of note, the list of characteristics used in this study contained many more behaviors (e.g., “cries often”) and preferences (e.g., “likes to play with tools”) than is typical in adult research (where most items are traits; e.g., “is sensitive to others’ needs” [38]); also, we included clothing choices and choices of toys (as these are important features of a child’s world and are frequently noted in studies of children’s gender development), even though such items would not typically be included in adult studies.

At the top of each page, participants were reminded of the instructions for the task: “Indicate how [desirable/typical] it is in American society for a 3-year-old [girl/boy] to possess each of the following characteristics.” Participants responded on a scale ranging from 1 (Not at all desirable/typical) to 9 (Very desirable/typical). We included two attention check items that embedded an instruction (e.g., “please select 9 so that you say strongly agree”) within our typical question frame (e.g., “How desirable is…”), to ensure that participants were paying adequate attention.

At the end of the study, participants were asked to identify themselves as Male or Female or say that they Preferred not to Answer. Participants also identified their race (Black, Asian, American Indian/Alaska Native, Hawaiian Native/Pacific Islander, White, Two or more Races, Prefer not to answer). Participants had the option to skip any questions without penalty. At the end of the study, participants were fully debriefed and compensated.

### Results

#### Data management

As outlined in our preregistration (https://osf.io/za6ah/), participants who responded to < 80% of the questions (*n* = 45) were excluded. Any participant (*n* = 28) who failed any one attention check question was also excluded. This left us with a final *N* = 562. Participant race was self-reported as follows: White (80%), Black (7%), Other (7%), Asian (3%), American Indian (2%), Hawaiian Native/Pacific Islander (1%); 50% self-reported as male and 50% as female.

#### Analyses

The primary goal of Experiment 1 was to identify characteristics (i.e. traits, behaviors, and preferences) that were gender-stereotypical for young boys and girls; our intention from the outset was to use these characteristics in constructing vignettes for Experiments. 2 and 3 that would reflect gender stereotypic and counterstereotypic children. Specifically, we wanted to identify descriptive characteristics for each gender, in order to create vignettes that were matched on typicality. More importantly, we sought to identify which characteristics were prescriptive and proscriptive for girls and for boys.

We began by identifying characteristics that were descriptive for boys and girls. To do so, for each characteristic, we conducted an independent samples *t*-test comparing ratings of typicality (i.e. our descriptive scale) for girls to ratings of typicality for boys; we then calculated Cohen’s *d* in order to determine the effect size for each descriptive stereotype. In keeping with our preregistration (https://osf.io/q63ae/) and prior research [38], we classified characteristics as descriptive for a particular gender if they were rated above ‘6’ for typicality for that gender on our descriptive scale, with a |*d*| > .4 when comparing ratings between the two genders. Table 1 shows the descriptive characteristics for boys and girls. There are several findings of note. First, for boys, the vast majority of descriptive characteristics were behaviors and preference. In contrast, half of the descriptive characteristics for girls were traits, and there were a large number of appearance-relevant characteristics. Second, nearly twice as many characteristics met the descriptivity threshold for boys (*n* = 19) than for girls (*n* = 10). Third, 80% of descriptive characteristics for girls were also either prescriptions for girls or proscriptions for boys, while only 57.8% of male-descriptive characteristics were also pre-/proscriptions. Fourth, on the whole, the characteristics that were descriptive of boys were quite negative; more than half of the characteristics had desirability ratings lower than the midpoint of the scale, and the average desirability rating of these descriptive characteristics was similarly below the midpoint of the scale (*M* = 4.95, *SD* = .87). In contrast, the descriptive characteristics for girls were quite positive: no characteristic was below the midpoint of the desirability scale, and the average desirability was quite high (*M* = 6.53, *SD* = .81).

**Table 1:** Descriptive characteristics for girls (left) and boys (right).

Characteristic	*d*	Characteristic	*d*
**Enjoys wearing skirts and dresses**	-2.59	**Plays with trucks**	2.06
**Likes princesses**	-2.35	**Likes to pretend to be a soldier**	1.96
**Loves pink**	-2.13	**Handsome**	1.74
**Wears Tutus**	-2.03	**Likes superheroes**	1.61
**Likes to play with dolls**	-1.48	**Likes to play with tools**	1.37
**Pretty**	-0.61	**Loves to get dirty**	1.16
**Gentle**	-0.55	**Dirty**	0.998
Caring	-0.55	Rowdy	0.93
Emotional	-0.5	**Has unbrushed hair**	0.76
**Sensitive**		**Has a big appetite**	0.7
		Messy	0.61
		Has bruised knees	0.6
		**Adventurous**	0.959
		Wears clothes that don't match	0.53
		Sometimes hits others	0.53
		Doesn't wait turn	0.46
		Interrupts others	0.44
		Refuses to pick up	0.43
		**Likes playing outside**	0.42

Note: Positive *d* indicates characteristic was rated as more typical for boys and a negative *d* indicates characteristic was rated as more typical for girls. Bold indicates that the characteristic was prescriptive for that gender and/or proscriptive for the opposite gender.

Next, we identified prescriptive and proscriptive characteristics for each sex by comparing ratings of desirability (i.e. our prescriptive/proscriptive scale) for girls to ratings of desirability for boys; again, we calculated Cohen’s *d* in order to determine the prescriptive and proscriptive stereotype gender effect sizes for each characteristic. We identified each characteristic as prescriptive for a particular gender if it was rated above a ‘6’ on our desirability scale and there was a |*d*| > .4 when comparing ratings for girls vs. boys. Table 2 shows the prescriptive characteristics for boys and girls. Similarly, characteristics were labeled as proscriptive for a particular gender if they were rated at or below a ‘4’ on our desirability scale and there was a |*d*| > .4 when comparing ratings for girls vs. boys. There were relatively few proscriptive characteristics for girls; in fact, unlike for adult women ([38]; see Table 2), we only found three proscriptive traits for girls (“is dirty,” “has unbrushed hair,” and “likes to pretend to be a soldier”). However, for boys, we found 9 proscriptive traits (Table 2).

**Table 2:** Prescriptive and proscriptive characteristics for boys and girls.

Characteristics					
Girls			Boys		
Prescription	De- *d*	Pre- *d*	Prescription	De- *d*	Pres- *d*
**Enjoys wearing skirts and dresses**	-2.6	-1.92	Handsome	1.74	1.51
**Likes to play with dolls**	-2.03	-1.69	Plays with trucks	2.06	1.31
**Likes princesses**	-2.34	-1.49	Likes to play with tools	1.37	1.17
**Loves pink**	-2.13	-1.37	**Likes to pretend to be a soldier**	1.96	1.14
**Wears Tutus**	-2.03	-1.34	Loves sports	1.09	0.87
**Likes to wear nail polish**	-1.68	-1.17	Likes superheroes	1.61	0.86
**Pretty**	-1.48	-1.15	Has a big appetite	0.7	0.66
Graceful	-0.73	-0.69	Strong	0.31	0.59
Helps mom bake	-0.57	-0.57	Tough	0.43	0.56
Gentle	-0.61	-0.56	Adventurous	0.6	0.54
Sweet	-0.17	-0.41	Loves to get dirty	1.16	0.54
Sensitive	-0.5	-0.41	Brave	0.31	0.52
			Likes playing outside	0.42	0.48
Proscriptive	De- *d*	Pro- *d*	Proscriptive	De- *d*	Pro- *d*
**Likes to pretend to be a soldier**	1.96	1.14	**Enjoys wearing skirts and dresses**	-2.6	-1.92
Has unbrushed hair	0.76	0.55	**Likes to play with dolls**	-2.03	-1.69
Dirty	1	0.4	**Likes princesses**	-2.34	-1.49
			**Loves pink**	-2.13	-1.37
			**Wears Tutus**	-2.03	-1.34
			**Likes to wear nail polish**	-1.68	-1.17
			**Pretty**	-1.48	-1.15
			Fragile	-0.56	-0.55
			Pays attention to what other people are wearing	-0.74	-0.43

Characteristics are listed in order of largest prescriptive/proscriptive *d* to smallest. Positive *d* indicates characteristic was rated as more typical, desirable or forbidden for boys. Negative *d* indicates characteristic was rated as more typical, desirable or forbidden for girls. Descriptives that were prescriptive for one gender and proscriptive for opposite genders appear in bold.

Finally, we developed a list of ungendered traits such that ratings were as close to ‘5’ as possible on the descriptive and prescriptive/proscriptive scales, and such that no |*d*| was greater than .2 when comparing boys to girls; this table is available in the Supplemental Online Materials ([S1 Text]; [Gender Backlash Manuscript Supplementary Materials.docx]). Of note—and perhaps surprisingly—a number of characteristics that are frequently discussed in the popular media as being gendered, were actually quite neutral in our study (e.g., bossy, anxious, energetic, disobedient, self-reliant).

### Experiment 1 discussion

In Experiment 1, we explored the content of adults’ gender stereotypes for three-year-old boys and girls. To do this, we asked participants to rate the typicality and desirability of characteristics for boys and girls. Consistent with the adult literature, we found a large number of prescriptive and descriptive traits for both boys and girls. In an interesting divergence from the adult literature, we found relatively few proscriptive traits for children, particularly for girls (indeed, there were only three: dirty, has unbrushed hair, and likes to pretend to be a soldier). The paucity of proscriptive characteristics for girls may suggest that adults are somewhat more tolerant of deviations from gender-stereotypical behavior from girls than they are from adult women or from young boys. However, such interpretations should be made with caution—as described earlier, our initial list of characteristics differed substantially from the lists typically administered to assess stereotypes about adults. More generally, even comparisons between the number of male vs. female proscriptions in our dataset should be done with caution; for example, more than half of proscriptions were about the child’s appearance; however, an inspection of the list of characteristics tested shows that we tested more items related to girls’ appearance than to boys’, and this could have caused such an asymmetry. Thus, while suggestive, we urge caution in interpreting the relative lengths of the lists of descriptive, prescriptive, and proscriptive characteristics.

Most importantly, we were able to use these ratings to develop a list of characteristics that were prescriptive and proscriptive for boys and girls, and these characteristics formed the basis of our stimuli in Experiments 2 and 3.

### Experiments 2 and 3

The purpose of Experiments 2 and 3 was to test for evidence of backlash against stereotype-violating children. Experiment 3 is a direct replication of Experiment 2: Conducting direct replications can increase the precision of effect estimates, while also allowing us to assess the robustness of our main findings [63–65]. Because the methods and analyses were identical across experiments, and the findings highly similar, we report Experiments 2 and 3 together, and make all materials available online on the Open Science Framework (OSF; Exp 2: https://osf.io/q63ae/; Exp. 3: https://osf.io/pwnxj/).

In Experiments 2 and 3, we constructed 'feminine' and 'masculine' vignettes (described below), and paired them with preschool applications for boys and girls. Thus, we orthogonally manipulated applicant sex (male vs. female) and vignette type ('masculine' vs. 'feminine'), such that participants could see a stereotype-conforming girl (feminine girl), a stereotype conforming boy (masculine boy), a stereotype-violating girl (masculine girl), or a stereotype-violating boy (feminine boy). We refer to these vignettes as 'feminine' and 'masculine' for ease of readability. However, from a theoretical perspective, it might be more appropriate to think of the 'masculine' vignettes as instead being ‘unfeminine’ (in that they contain traits that are descriptive of boys, prescriptive for boys, and proscriptive for girls). Similarly, it might be more appropriate to think of the 'feminine' vignettes as being ‘masculine’ (because they contain characteristics that are descriptive of girls, prescriptive for girls, and proscriptive for boys).

The goal of these experiments was to test for evidence of backlash against gender nonconforming three-year-olds. At the outset, based on the adult literature, we had two main hypotheses. First, consistent with past literature showing that gender stereotype-violating individuals are reliably rated as less likeable than similar stereotype-conforming targets (see [39,66]), we hypothesized that we would find evidence of gender backlash when measuring an applicant’s likeability. In particular, we expected that stereotype-violating individuals would be rated as less likeable than identical stereotype-conforming individuals. In this experimental context, such an effect would manifest as an interaction of sex and vignette on ratings of likeability. We also hypothesized that, consistent with the literature on backlash against adults (e.g., [25,38,66]), because children’s competence was clearly communicated, there would be no effect of applicant sex or vignette type on perceptions of children’s competence. For the remaining outcome variables, there was not sufficient pre-existing literature to warrant specific *a priori* predictions. Therefore, we examined whether there were effects of the target child’s sex, vignette type, and/or their interaction on ratings of admissibility to preschool (our conceptual analogue of “hireability” in the adult literature), the amount of scholarship offered to the child, willingness to interact with the child, moral outrage regarding the child, and perceptions of the child’s parents.

### Method

#### Timeline and preregistration

We collected data for Experiment 3 in June of 2017, 2 month after completing data collection for Experiment 2 (which was collected in April, 2017), and thus Experiments 2 and 3 were preregistered separately (https://osf.io/zudta/ and https://osf.io/2yu3b/).

#### Participants

Data were collected from 697 (Exp. 2) and 731 (Exp. 3) adults from the United States, aged 18 years or older, on Amazon Mechanical Turk. Recruitment procedure, criteria, and compensation were as in Exp. 1.

#### Material and procedure

Participants completed the task independently on their own computer using the web survey program Qualtrics; we used Turk Prime to manage participant recruitment [61]. The study took an average of 11.5 minutes to complete, and included 57 questions and 2 attention checks. Participant consent was obtained as in Experiment 1.

Participants were instructed that they would read an actual application “of a child who recently applied to a daycare/preschool with limited space available,” and that they were going to be asked to evaluate and provide feedback for the child. As in previous work, we noted that that their honest opinion was requested (adapted from [67]). To ensure comprehension of the task at hand, these instructions were broken into four paragraphs; after each paragraph, the participant was asked to answer a comprehension question (https://osf.io/q63ae/ for full materials). Participants who did not answer a comprehension question were reminded to answer it; those who responded incorrectly to the comprehension questions were excluded (described below).

After participants demonstrated their comprehension of the task at hand, they saw the following instructions: “Below is the **actual application of a child who recently applied to a daycare/preschool with limited space available.** Please read the applicant’s profile carefully. You will be asked questions about the child.” Participants were then randomly assigned to view one of four profiles as part of our 2(sex: boy, girl) x 2(vignette: masculine, vignette) between-subjects design. Participants either learned (through the child’s name, pronouns, and noted sex) that the child was a boy or a girl. We selected the names (Jennifer vs. Jonathan) because they had been previously rated similarly on a host of measures [68], allowing us to focus on effects of sex (and not of, for example, name-based biases). Participants were then exposed to vignettes (supposedly constructed from note from a previous teacher) demonstrating the child’s behavior as either 'feminine' or 'masculine'.

We designed our vignettes by relying on the norming data from Experiment 1. First, both vignettes contained the neutral characteristic “clever” (descriptive *d* = -.07; pre/proscriptive *d* = .08) The 'feminine' vignette also contained 1 female-descriptive characteristic (“sensitive”), 1 female-prescriptive and male-proscriptive characteristic (“loves playing with dolls”), and 1 male-proscriptive characteristic (“pays attention to the outfits that other people are wearing”). The 'masculine' vignette contained 1 male-descriptive characteristic (“strong”), 1 male-prescriptive and female-proscriptive characteristic (“loves pretending to be a soldier”), and 1 female-proscriptive characteristic (“has unbrushed hair”). Within the constraints of the characteristics rated in Experiment 1, we constructed these vignettes to be as closely matched as possible thematically (soldiers vs. dolls; sensitive vs. strong; unbrushed hair vs. pays attentions to outfits). We also aimed to equate the vignettes as much as possible based on the ratings provided in Exp. 1. To do this, we computed the average typicality and desirability ratings for the characteristics included in each vignette; this allowed us to ensure that one vignette was not wildly more positive or normative than the other. The mean typicality and desirability of the traits in the 'feminine' vignette (5.12_typical_, 5.14_desire_) was similar to the 'masculine' vignette (5.04_typical_, 5.05_desire_); similarly, the mean effect size (for boy vs. girl comparisons from Exp. 1) for the descriptive and pre/proscriptive traits was comparable for the 'feminine' (1.09_descriptive_, .85_pre/proscriptive_) and 'masculine' (.85_descriptive_, .76_pre/proscriptive_) vignettes. The vignettes were as follows:

“[Jennifer/Jonathan] is a healthy three-year-old [girl/boy] who [absolutely loves playing with dolls]/[loves pretending to be a soldier]. [He/She] frequently [pays attention to the outfits that other people are wearing]/[has unbrushed hair]. When asked to list the two adjectives that best described [Jennifer/Jonathan], the adjectives that [his/her] previous teacher provided were “[sensitive]/[strong]” and “clever.””

After viewing the vignettes, participants evaluated the applicant by responding to questions on a likert scale ranging from 1 (not at all) to 7 (very much). Within each page, the order of the scales and questions within scales were randomized, although the order of pages within the survey was fixed. On the first page, participants were asked about the applicant’s: likeability (*n* = 4 questions; Cronbach’s alpha = .88_Exp2_; .89_Exp3_; e.g., “How much do you think this child’s teachers would like this child?”), competence (*n* = 5 questions; Cronbach’s alpha = .94_Exp2/3_; e.g., “To what extent do you think this child would do well in this school?”), and “admissibility” (*n* = 3 questions; Cronbach’s alpha = .85_Exp2_; .84_Exp3_; e.g., “How likely do you think it is that the child was actually accepted into the daycare to which they applied?”).

On the next page, they were asked how much of a scholarship they would give to the applicant: “If you had the option of giving this applicant a scholarship to attend daycare/preschool, how much of a scholarship would you give?”, and they indicated their answer on $0-$1000 sliding scale ($0 = no scholarship; $1000 = full scholarship). Participants also made judgments about their willingness to interact with the applicant (*n* = 3 questions; Cronbach’s alpha = .79_Exp2_;.76_Exp3_; e.g., “If you had a three year old child, how likely would you be to invite this child over for a playdate?”), their moral outrage at the applicant (*n* = 3 questions; Cronbach’s alpha = .94_Exp2_; .93_Exp3_; e.g., “To what extent would you describe this child’s behavior as morally wrong?”), and their perceptions of the applicant’s parents (*n* = 6 questions; Cronbach’s alpha = .82_Exp2_; .84_Exp3_ ;e.g., “To what extent did you think that this child’s parents were doing a good job raising them?”).

Next, participants were asked to guess the purpose of the study (“Please summarize, to the best of your ability, the purpose of this study”) and were then presented with two measures of their recall of the applicant’s profile (“What was the applicant’s gender? [boy, girl, do not recall]”; “What was the applicant's age? [3, 4, do not recall]”). They were then asked to freely respond to the following question: “What do you remember about the applicant’s play-time activities?”.

On the next page, participants were asked to make inferences about the child’s gender and sexuality (Antill, 1987; Martin, 1990). First, participants were asked: “If you had to guess, do you believe that this child is gay/lesbian? [Yes, No, Not Possible to Tell]”. Second, participants were asked: “If you had to guess, do you believe that this child is transgender? [Yes, No, Not Possible to Tell]”.

Next, participants continued to the Participant Characteristics portion, for which they saw the following: “Now, you will answer questions about yourself.” Participants completed the Modern Sexism Scale 1–7 (e.g., “Discrimination against women is no longer a problem in the United States.”; 1 = not at all; 7 = very much; [69]), an experience with infants and kids scale (e.g., “How much experience have you had with INFANTS (18 months or under) in the last 5 years?”; [45]), and the same demographic questionnaire (asking participants to self-report race and gender) from Experiment 1. They were also asked to identify their political orientation on a 7-point scale, their teacher status (Teacher, Never Teacher), and their parenting status (Parent, Never Parent).

After completing all evaluation measures, participants were asked to note any questions, comments, or concerns they had about their participation or the task itself. At the end of the study participants were debriefed and fully compensated.

### Results

#### Data management

We excluded a total of 59_Exp2_/95_Exp3_ participants, leaving final *Ns* = 595_Exp2_/636_Exp3_. As planned in our preregistrations (https://osf.io/zudta/ and https://osf.io/2yu3b) participants were excluded for the following reasons: failure to complete at least 80% of the experiment (*n* = 21_Exp2_/36_Exp3_), failure to demonstrate understanding of experimental instructions as indicated by at least 1 incorrect response for our task-comprehension questions (*n* = 25_Exp2_/26_Exp3_), failure of one or more attention checks (*n* = 12_Exp2_/29_Exp3_). One participant in Experiment 2 was excluded because they reported not having been able to see our experimental vignette; four participants in Exp. 3 were excluded for guessing the purpose of the study. There were no trial-level exclusions, and recall of vignettes was strong: 98.9% of participants recalled the applicant’s Sex and 98.1% of participants recalled the applicant’s age (recollection of sex/age was not an exclusion criterion).

Our participants were 78_Exp2_/75.8_Exp3_% White (8.1_Exp2_/7.7_Exp3_% Black; 5.9_Exp2_/7.9% Asian; <5% each “American Indian/Alaska Native”, “Hawaiian Native/Pacific Islander”, “Other/prefer not to answer”, and “Two or more races”). We had not specified in our preregistration for Experiment 2 how we would analyze race, although we did in Experiment 3: Consistent with previous research (e.g., [70]), we recoded race as “White” vs. “Person of color,” and omitted participants who selected ‘Other/prefer not to answer’ from *only* analyses that include race as a factor. Among our participants, 51.9_Exp2_/53.6_Exp3_% of identified themselves as Female (<1% failed to identify or identified as “Other”). As planned in both Experiments, we also omitted participants who identified their gender as ‘Other/prefer not to answer’ *only* from analyses that included participant gender as a factor. Just over 52_Exp2_/50_Exp3_% of participants identified themselves as parents, while 47.9_Exp2_/49.8_Exp3_% identified themselves as “Never a parent”; 13.1_Exp2_/14.6_Exp3_% of our participants identified as teachers. For 97_Exp2_/96.3_Exp3_% of participants, English was a first language, and 95.6_Exp2_/94.1_Exp3_% of participants reported having been born in the United States. On a 1–7 scale (1 being extremely liberal, 7 being extremely conservative), our participants had an average score of 3.42_Exps2and3_; on a scale of 0–10 (0 being no contact with infants/children; 10 being large amounts of contact with infants/children), our participants had an average score of 5.62_Exp2_/5.74_Exp3_; on a scale of 1–7 (1 reflecting low levels of modern sexism and 7 reflecting high levels of modern sexism), our participants had an average score of 3.14_Exp2_/3.21_Exp3_ on the Modern Sexism Scale.

We constructed scales in keeping with our preregistrations by taking the average rating for the items within each scale (reverse coding when relevant); we did not remove any outliers.

#### Analyses

Our analyses are as planned in our preregistrations (https://osf.io/zudta/ and https://osf.io/2yu3b), unless otherwise noted. Our main goal was to test for backlash against preschool applicants. If children experience gender backlash, then stereotype-violating children whose sex differs from their gendered actions (i.e. girls who are described as masculine, and boys who are described as feminine) will be rated more negatively than the identical stereotype-conforming children (i.e. girls who are described as feminine, and boys who are described as masculine). To this end, we submitted each dependent variable to a 2 (applicant Sex) X 2 (vignette type: 'feminine', 'masculine') ANOVA, in order to detect an interaction of Sex and vignette. As planned at the outset, we explored the direction of effects using Tukey’s HSD, and used a critical cut-off of .05 for these analyses, since Tukey’s HSD is a conservative test that adjusts for multiple comparisons.

Consistent with previous work in this area (e.g., [38]), in Experiment 2 we did not establish a plan for controlling for false discovery rate (FDR), instead specifying in our preregistration an alpha of 0.05 for each of our analyses. However, given the large number of primary and secondary effects included in our design, we invited a statistician co-author to join our research team, and made the decision (after collecting Experiment 2 data but prior to preregistering or collecting data for Experiment 3) to control our Type-I error rate using the Benjamini-Hochberg (B-H) criteria to determine significance [71–72]. As such, we deviated from our preregistration for Experiment 2 (but not for Experiment 3) in this regard. The B-H method of controlling the FDR is straightforward to implement, and has previously been proposed as a useful statistical tool in the educational and psychological literatures [73–75]. Specifically, we tallied the number of *p-*values for each Experiment that we planned to calculate in our two sets of analyses: (1) our main analyses, which tested only for the effects of Sex and vignette (and their interaction) on ratings (*n* = 21); and (2) our analyses that aimed to detect a three-way-interaction of Sex, vignette, and Participant Characteristics on ratings (*n* = 49). For each set of analyses, we divided .05 by the number of analyses (e.g., .05/21) and constructed ordered quantiles such that the upper bound of quantile *n* was (.05/21)*n (e.g., the upper bound of the 1st quantile was .00238, for the 2nd it was .0047, for the 21st it was .05). We then ordered the *p*-values that resulted from our analyses from smallest to largest, and compared each *p*-value to its rank-matched quantile (such that *n*th smallest *p*-value was compared to the critical value represented by the upper bound of the *n*th quantile). As such, we treat only those analyses for which the *p*-values fell below the Benjamini-Hochberg threshold as meeting our significance criteria. For the sake of comparison to previous literature (which typically has used a cutoff of .05), we report additional *p*-values in the SOM [S1 Text], and make our dataset, analysis code, and analysis outputs freely available for this and all analyses analysis at our OSF site (https://osf.io/pwnxj/)

In our preregistration for Experiment 3, we described our criteria for replicating a particular effect across experiments as being the overlap of 95% CIs. As a supplement, we added un-preregistered analyses to estimate effect sizes across Experiments 2 and 3 using the single paper meta-analysis (SPM) approach proposed by McShane and Böckenholt [76]. SPM is designed to aggregate information from multiple studies produced using identical sets of conditions and outcomes to estimate a single effect size. Given the number of variables in our study, SPM will often be preferable for identifying our best estimate of true effect sizes, in place of providing intervals from both experiments in the main text (these are available in our analyses and code; in no case does SPM give meaningfully different outcomes than emerged from a simple interval comparison).

**Fig 1:**
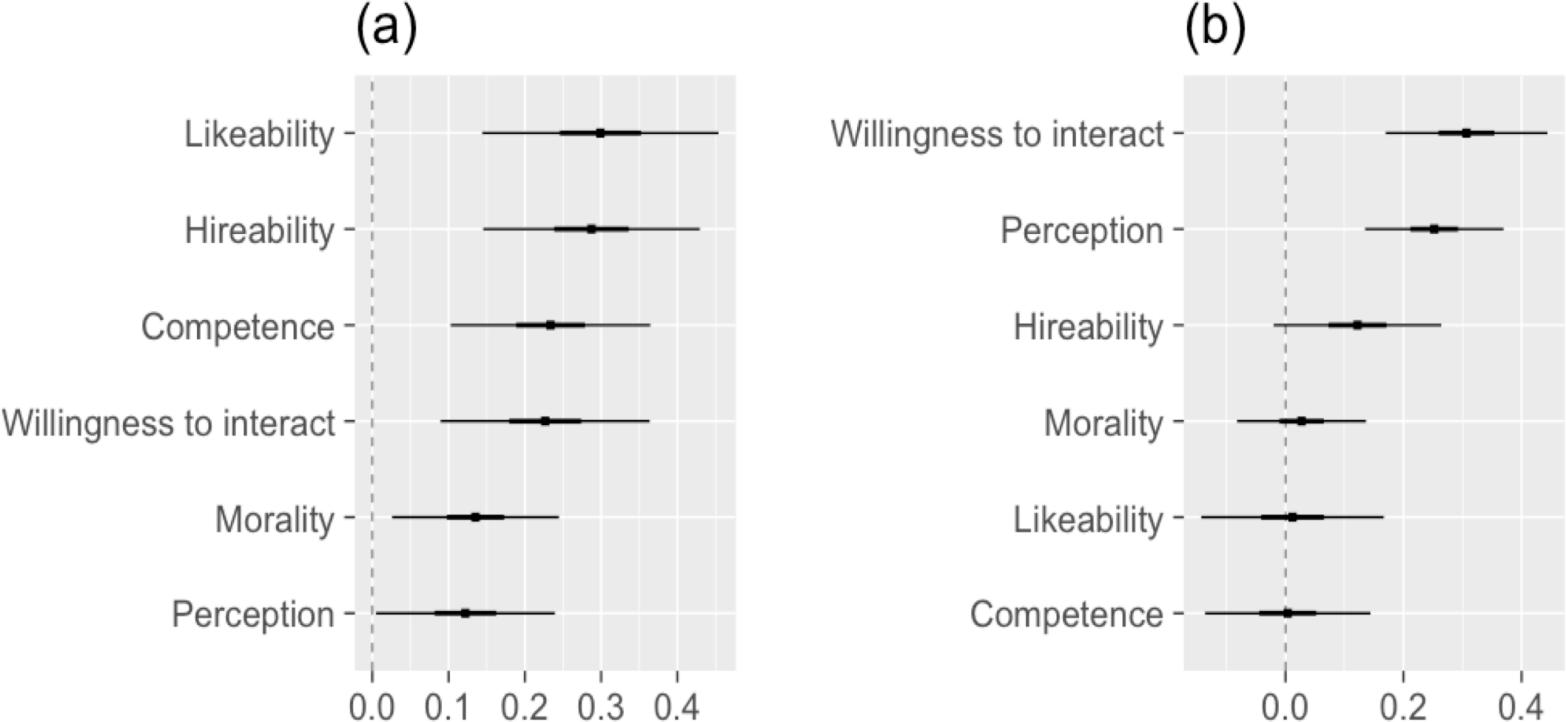
Single paper meta-analysis (SPM) estimated change in response for (a) girls (relative to boys) and (b) 'feminine' vignettes (relative to 'masculine'), shown with 50% and 95% confidence intervals. *Scholarship conferral* is not shown as it was measured on a different scale.

While not relevant to our question of backlash, we first explored the presence (or absence) of simple effects of sex and vignette type. Sex and vignette-level analyses revealed an overall preference for female applicants and, to a lesser extent, 'feminine' vignettes. Fig 1 highlights average sex-differences calculated using SPM. In Fig 1, each point roughly reflects a weighted average of the sex- or vignette-level differences observed in both Experiments 2 and 3, with weights proportional to the number of respondents. At the 0.05 level, the six dependent variables shown in Fig 1 differed by sex, with four differences (Likeability, Admissibility, Competence, Willingness to interact) significant after controlling for FDR using the B-H adjustment. In other words, girls were viewed as more likeable, hireable, competent, and were more likely to inspire adults to interact with them relative to identical boys, regardless of the child’s behavior. Ratings on our Willingness to interact and Parent Perception scales were also significantly higher for 'feminine' vignettes than for 'masculine' vignettes.

**Fig 2:**
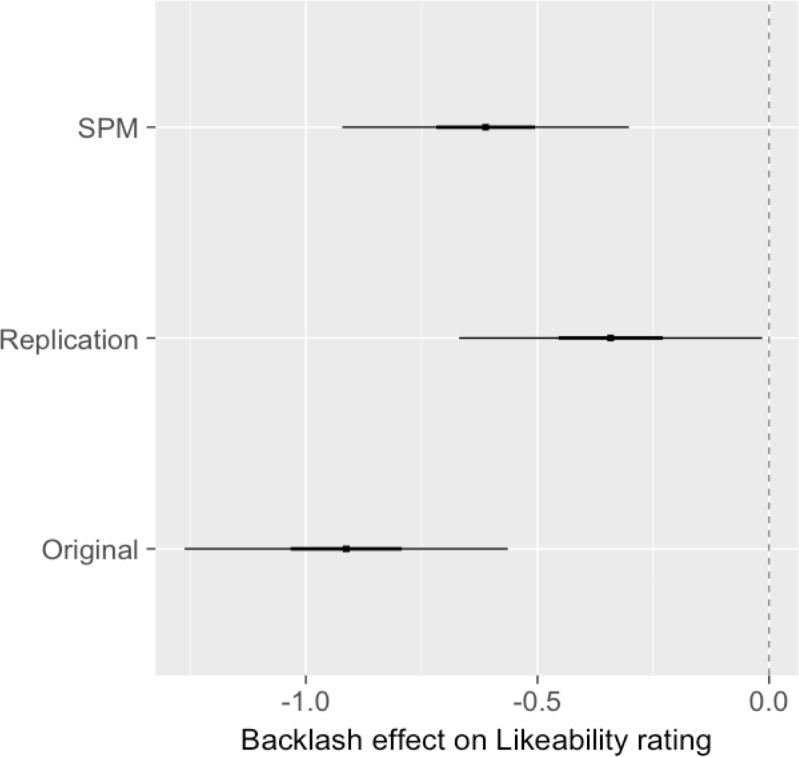
Likeability backlash effect estimates in Experiment 2 (original) and 3 (replication), as well as the single paper meta-analysis (SPM) estimate of the true effect (50% and 95% intervals also shown). The scale of the response corresponds to the average change in Likeability rating when assigned a vignette of the opposite gender, relative to expectations given sex and vignette averages alone.

Our primary measure of interest was likeability—as has been previously noted (Moss-Racusin, 2014), likeability has been identified as the most critical and consistent indicator of backlash against stereotype violators. Consistent with this, we found interactions of sex and vignette in Experiment 2 (*p* < .0001, η^2^ = .04) and Experiment 3 (*p* = .04, η^2^ = .007), the former of which met our B-H cutoff for significance. The magnitudes (with 50% and 95% intervals) for backlash (likeability) are visualized in Fig 2, which shows estimates from Experiment 2 and Experiment 3, as well as the SPM estimate. As described in our preregistration for Experiment 3, the overlapping 95% CIs met our criteria for replication of findings across experiments.

For visualization purposes, differences in Fig 2 reflect the change in average likeability score for each sex when assigned a vignette of the opposite gender (about 0.6 lower, averaged across the two experiments), relative to what we’d expect given sex and vignette averages alone. If there were no likeability backlash effects, we’d expect estimated differences near 0. The directions of the estimates in Fig 2 reflect the reference group choices for sex and vignette; our findings can also be interpreted to mean that children whose vignette matched their sex scored about 0.6 higher on the likeability scale.

We next used Tukey’s HSD to conduct pairwise comparisons for the four cells of our design (Boy vs. Girl x 'Feminine' vs. 'Masculine'). Analyses for each Experiment separately are available in the SOM [S1 Text] and at our repository linked to above; because the combined dataset likely provides our best estimation of the size and direction of our effects, we report Tukey’s HSD for the combined dataset here. Stereotype-conforming girls were rated as significantly more likeable than stereotype-violating girls (*p* = .001, *d* = .30) and than stereotype-violating boys (*p* < .0001, *d* = .55). Also consistent with backlash against stereotype-violators, we found that stereotype-conforming boys were rated as significantly more likeable than stereotype-violating boys (*p* = .004, *d* = .27). Finally, consistent with evidence from the adult literature [77], there was evidence that girls were, overall, more likeable than boys: stereotype-conforming girls were rated as more likeable than stereotype-conforming boys (*p* = .002, *d* = .28), and stereotype-violating girls were rated as more likeable than stereotype-violating boys (*p* = .008, *d* = .26).

As has been previously reported in adults, measures of Competence typically do not show evidence of backlash in cases where competence was clearly demonstrated in each applicant profile [25, 38, 66]. Consistent with this, we found no backlash effects for competence (all unadjusted *p*>.05, see SOM [S1 Text] for full statistical reporting). We next assessed ratings of admissibility. Diverging from the adult literature [34], we found no evidence of admissibility backlash in either of our experiments (all unadjusted *p* > .05).

We next considered several variables that were new to our study; because these variables had not previously been studied in adult backlash work, we did not make strong *a priori* predictions about them, but instead included them in our study on the logic that they would be useful for understanding the ways in which backlash against stereotype-violating preschoolers might manifest. Across both experiments, there were was no evidence of backlash for Scholarship Conferral, Moral Outrage, Willingness to Interact, or Perceptions of Parents (all unadjusted *p* >.05).

Thus far, our data revealed evidence of backlash against stereotype-violating boys and girls with respect to the critical likeability outcome. However, Experiment 2 revealed multiple examples of backlash effects interacting with Participant Characteristics (see SOM [S1 Text]). We had not anticipated this possibility at the outset of our study, largely because participant-level demographics rarely impact backlash against stereotype-violators (e.g., [25, 39, 66]). Thus, we deviated from our preregistration for Experiment 2 (although not from our preregistration for Experiment 3) in another important way, by analyzing the effects of Participant Characteristics (Race, Male/Female, Teacher Status, Parent Status, Political Orientation, Modern Sexism Score) variables which initially showed no initial evidence of backlash (Moral Outrage, Competence, Admissibility, Perceptions of Parents, Willingness to Interact, and Scholarship Conferral) in addition to the variable which did show evidence of backlash (Likeability). These analyses were post-hoc and exploratory for Experiment 2, and confirmatory for Experiment 3.

We constructed models estimating Moral Outrage, Competence, Admissibility, Perceptions of Parents, Likeability, Willingness to Interact, and Scholarship Conferral from Sex, vignette, each Participant Characteristic, and their interaction; for this analysis, we were primarily interested in the three-way interaction of Sex, vignette, and Participant Characteristic.

**Fig 3:**
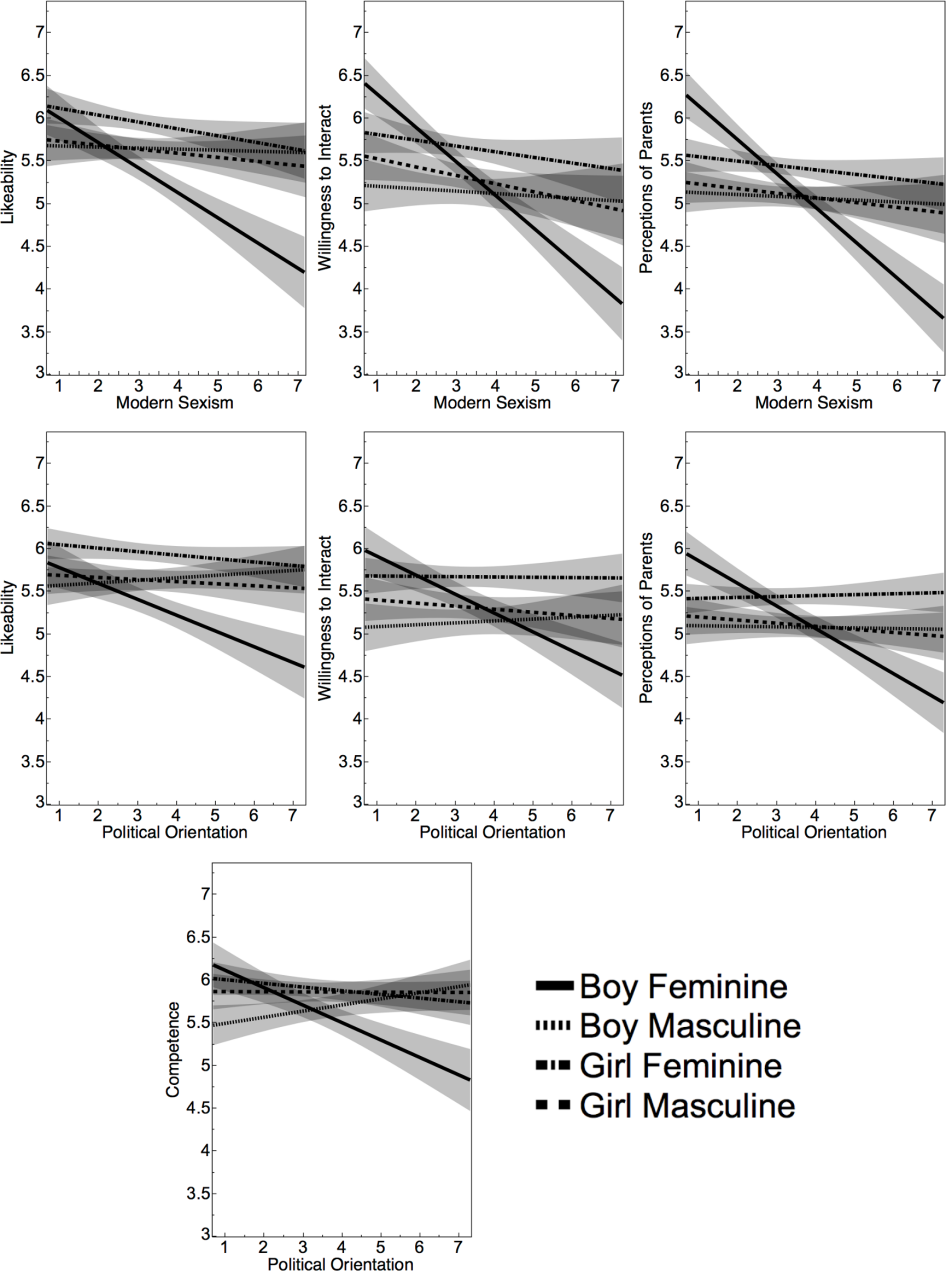
Participant characteristics * Sex * Vignette interactions. Higher values on the x-axis indicate higher levels of political conservatism or sexism. Shading represents fit confidence.

Using SPM and accounting for FDR using the B-H criteria, seven sex*vignette*Participant Characteristic interactions emerged when combining Experiments 2 and 3. Each of these three-way interactions are shown via lines of best fit in Fig 3 (shown with fit uncertainty). For all of these interactions, the direction was such that backlash effects on likeability ratings were larger for politically conservative individuals and for individuals who scored higher on the modern sexism scale. By and large, these effects seemed driven by ratings of feminine boys, shown in Fig 3 using the dark, undotted lines. The interactions depicted include sex*vignette*sexism (with Likeability: *p* = .005, η^2^ = .007; Perceptions of Parents: *p* < .0001, η^2^ = .015; Willingness to Interact: *p* < .0001, η^2^ = .013), and sex*vignette*political orientation (shown with Likeability: *p* = .005, η^2^ = .007; Perceptions of Parents: *p* < .0001, η^2^ = .017; Willingness to Interact: *p* < .0001, η^2^ = .0096; Competence: *p* = .001, η^2^ = .009). For each of these seven interactions, corresponding intervals between Experiment’s 2 and 3 were near perfect overlaps (see SOM [S1 Text]).

Using the B-H criteria, there were no 3-way interactions for any dependent variables when participant gender, participant race, experience with children, participant parent-status, or participant teacher-status were added to the model (but see our full dataset/analyses and SOM [S1 Text] for cases meeting traditional significance thresholds, but not B-H thresholds). As reported in our SOM, there were also no interactions in models estimating scholarship conferral, admissibility, or moral outrage.

We also measured whether participants believed that the applicant was (a) transgender and/or (b) gay/lesbian. The analyses below were exploratory in Experiment 2 and confirmatory in Experiment 3. While the vast majority of participants either said that the applicant was not transgender (40.7_Exp2_/40.9_Exp3_%) and not gay/lesbian (33.4_Exp2_/34.1_Exp3_%) or that it was not possible to tell (Transgender: 58.3%_Exps2and3_, Gay/Lesbian: 61.8_Exp2_/63.5_Exp3_%), a subset asserted that the child was transgender (*n*_Exp2_ = 6; *n*_Exp3_ = 5) or gay/lesbian (*n*_Exp2_ = 28; *n*_Exp3_ = 15). Using Fisher’s Exact Test to compare rates of “yes” vs. “no” responses, we found that in Experiment 2, only feminine boys were ever described as transgender or gay (simulated *p*-values < 0.001). In Experiment 3, we similarly found that feminine boys were more likely to be described as transgender (*p* = .03) and gay (*p* < .001) compared to any other group. These data are consistent with at least two previous studies [55–56] in which stereotype-violating boys were more likely to be rated as gay than were stereotype-violating girls or stereotype-conforming individuals.

### Experiments 2 and 3 discussion

In Experiments 2 and 3, we asked whether there was evidence of backlash against gender stereotype-violating three-year-olds. We had four main findings. First, across several of our measures, girls were rated more favorably than boys, and 'feminine' vignettes were rated more favorably than 'masculine' vignettes. While these findings do not speak directly to gender backlash, they suggest that in our experimental context, there was some evidence of a “women are wonderful” effect [77]. Second, we found that, like adults, three-year-olds who violate gender stereotypes are rated as less likeable that three-year-olds who conform to gender stereotypes. Third, we found that, unlike in previous work (for a discussion, see [66]), political orientation and scores on the modern sexism scale are linked to backlash effects. Finally, we found that, across a host of measures, stereotype-violating boys were penalized the most.

## General discussion

The present study represents one of the first efforts to experimentally determine the content of gender-stereotypes for young children, and to test for evidence of backlash from adults against children who violate those stereotypes. To do this, in Experiment 1, we first measured adults’ beliefs about which characteristics are descriptive, prescriptive, and proscriptive for young boys and girls. We then used the characteristics that were rated as descriptive, prescriptive, and proscriptive for each sex to constructed short vignettes aimed at capturing 'feminine' and 'masculine' characteristics. In Experiments 2 and 3, we presented participants with these 'feminine' and 'masculine' vignettes, and, critically, manipulated whether the vignette was attributed to a three-year-old girl or a three-year-old boy. We then asked whether adults penalized gender-stereotype violating children.

Our main finding was that three-year-old children, like adults [25, 38], experience gender backlash from adults. Specifically, children who violate gender stereotypes were rated by adults as less likeable than their stereotype-conforming peers. Most striking was the difference in likeability between feminine boys and feminine girls: when displaying identical (feminine) characteristics, boys were rated as substantially less likeable than girls. While the size of the backlash effect on likeability was smaller in Experiment 3 than Experiment 2 (suggesting some attenuation towards the null), using both our preregistered criteria of overlapping confidence intervals and single paper meta-analytic techniques [76], evidence of gender backlash with respect to likeability persisted.

There were several other ways in which our findings for children mirrored previous research on backlash against stereotype-violating adults. First, we found evidence of backlash from adults against feminine boys. Both early work [52, 54–56] and more recent cross-cultural work (e.g., [78]) suggests that boys may be most likely to be penalized for violating gender stereotypes , and the current work supports this finding (but see [79]). Specifically, we found that feminine boys were rated as least likeable (relative to all other target children), and when backlash emerged, it appeared to be more extreme for feminine boys than for masculine girls. This extends previous work in a substantial way by demonstrating that in carefully controlled experimental contexts, feminine boys experience uniquely robust social penalties from adults.

Second, we found that, consistent with previous work [77], there was evidence of a “women are wonderful” effect in children: girls, and especially feminine girls, were generally rated more positively by adults than all other targets in our experimental context. In fact, this work shows the first (to our knowledge) evidence of a “girls are wonderful” effect, consistent with the established “women are wonderful” effect in the literature. Why might this be? There are at least three possibilities (not mutually exclusive with one another). One possibility is that, consistent with existing theories of the “women are wonderful” effect, in cases where existing power structure are not threatened by women, girls are evaluated positively [80]. In other words, because our target children were 3-year-olds, their characteristics may not have been viewed as potentially threatening to existing power structure, resulting in a “girls are wonderful” effect. A second possibility is that our data actually show a “boys are bad” effect [81]. Previous research has shown that when presented with another child’s ambiguous actions, school-aged children tend to assume boys to be bad [81]; consistent with this, more than half of the descriptive characteristics for boys found in Experiment 1 were negative (e.g., interrupts others, refuses to pick up), and, on average, boys’ descriptive characteristics were rated as undesirable. In contrast, none of the descriptive characteristics for girls were rated as undesirable. A final possibility is that the school-related framing of our study encouraged higher ratings for girls and femininity. Specifically, our experiment was framed in terms of preschool admissions, and previous work has shown that childhood educational spaces are typically perceived as feminine. For example, there is evidence of backlash against men who want to be teachers [42]. It may be the case that by describing the targets as preschool applicants, we established a context in which girls and feminine characteristics would be prioritized. To test this possibility, future researchers should test for evidence of backlash against children in contexts like competitive sports, where boys and masculine characteristics are traditionally more valued.

While we found evidence that stereotype-violating children, like adults, experience backlash from adults, our present study revealed findings that diverged in several important ways from the existing adult literature. First, we found no evidence of backlash in preschool admissions decisions; while there has been some evidence of economic and social penalties (i.e. backlash effects in “hireability”) in the adult literature, our child-relevant measure of “admissibility” did not elicit such backlash effects. This could be for a number of reasons: perhaps children truly do not experience backlash from adults in the form of hiring/admissions; perhaps our participants were hesitant to deny educational opportunities to young children (e.g., we had many participants who, in the comments section, advocated for universal admission to preschool—we assume it is unlikely that they would also advocate that all people should be hired for all jobs); perhaps preschool admissions (unlike hiring) doesn’t measure economic penalties; and/or perhaps individuals do not reason about school admissions to preschool in the same way they reason about hiring. For example, jobs are often thought to be earned and merit-based, while school admissions decisions (and perhaps especially preschool/daycare admissions decisions) may be viewed as less dependent on merit than potential, or may even be viewed as unrelated to the child’s traits, characteristics, and actions. Also, it is often only possible to hire a single individual for a job (making the hiring stakes very high; hiring one person entails *not* hiring others and therefore is a moment in which existing power structures can either be challenged or maintained) while typically multiple children are admitted to a particular daycare/preschool at a time (thus lowering the stakes of any individual admissions decision). Thus, future work should further explore whether backlash against stereotype-violating children can comprise economic penalties by exploring alternate operationalizations of “hireability” or additional economic consequences. More broadly, future work should consider alternative ways of assessing prejudice and discrimination targeting children rather than adults.

A second way in which our study differed from the studies of in which adults are the stereotype-violators was that we found several cases in which Participant Characteristics impacted the presences of backlash. Political Orientation and Modern Sexism Score both predicted backlash in the form of lower ratings for stereotype-violating targets on likeability, willingness to interact with the target child, and perceptions of the target child’s parents. Political Orientation also impacted evidence of backlash in ratings of competence. Specifically, our results revealed that higher levels of political conservatism and modern sexism were associated with more negative reactions to gender stereotype-violating individuals, and this effect appeared to be driven by stereotype-violating boys. These data were surprising for several reasons. In cases where adults violate gender stereotypes, there is little evidence that Participant Characteristics impact backlash [66]; also, there are rarely effects of backlash on ratings of an applicant’s competence [25]. From the present study, it is still unclear why we found effects of Participant Characteristics on backlash. One possibility is that these effects are present in adults, too, but previous studies lacked the power to detect them. For example, in the present study (with over 1200 participants across Exps. 2 and 3), our power was only .80 to detect the three-way sex*vignette*modern sexism score interaction on likeability; if we had had (as is typical) only 20–40 participants per cell, our power to detect such an effect would have been only .11-.17. A second possibility is that political orientation and modern sexism score predict backlash against children, but not adults. Because the present study only tested for backlash against children by adults, we cannot differentiate between these possibilities. Future work should thus recruit large samples and include assessments of similar stereotype-violating and stereotype-conforming adults and children within the same paradigm, in order to determine whether Participant Characteristics may also shape backlash against adults.

One additional finding worth considering is that, while the vast majority of participants did not infer that the children in our study were gay/lesbian or transgender, we did find that feminine boys were most likely to be assumed transgender and gay. Previous literature has suggested that adults sometimes make inferences about gender and sexuality on the basis of children’s violations of gender stereotypes, and that they are somewhat more likely to assume that stereotype-violating boys (relative to stereotype-violating girls) are gay [55–56]. Further, previous work has shown that acceptance of gender-stereotype violation in children is negatively correlated with homophobia [52]. While very few participants believed that the target child was gay/lesbian/trans*, our data are consistent with these previous studies showing that stereotype-violating boys may be more likely to be perceived as gay.

As this is one of the first studies to experimentally measure adults’ backlash against stereotype-violating children, there is substantial additional future work to be done. First, there is good reason to assess the presence of backlash against children both younger and older than those tested in our study. Critically, our study revealed neither the age of onset of backlash or adult-like backlash. In the interest of understanding the developmental trajectory of children’s experiences of gender backlash, future work should extend the age range of the child targets to include both younger (into infancy) and older (through adolescence). Second, future work should systematically investigate whether children themselves enact backlash against stereotype-violating children and stereotype-violating adults. Doing so will greatly expand our understanding of the ways in which gender stereotypes impact (and are policed by) even very young children. More generally, future work should directly investigate whether backlash reinforces gender stereotypes against children by exploring whether stereotype-violating kids display a fear of backlash, and whether this fear of backlash limits their future gender-nonconforming behavior the way it does for adults [60].

Because the present study opens up several productive future avenues for research, we want to highlight several methodological and analytical choices in the present study that we (a) believe strengthen our results and (b) hope future researchers adopt. First, by using experimental methods (as opposed to observational or correlational methods), we could manipulate both the sex of the target and the femininity/masculinity of the target’s characteristics, and could also maintain careful control over the target characteristics across conditions (such that the feminine boy displayed *literally* the same behaviors as the feminine girl); this allows us to draw relatively stronger conclusions about the causal effects of sex and masculinity/femininity on perceptions. Second, the use of a relatively larger sample size allowed us to avoid some of the risks typical of underpowered studies. Third, our use of preregistration allowed us to be clear about the confirmatory vs. exploratory nature of each of our analyses; given the density of this dataset and the complexity of reasoning about higher order interactions, internal replications, etc . . ., the use of preregistered analyses (and the ability to clearly describe moments when we deviated from our preregistration) promotes greater confidence in the findings we present. Fourth, by controlling for the False Discovery Rate, we have increased assurance that our significant findings represent a true, underlying signal. Finally, we included both an initial experiment and a replication, and used single paper meta-analytic techniques [76] to aggregate information across two datasets while accounting for variation within and between experiments. This final step allows us to estimate aggregated effect sizes with the appropriate precision.

To conclude, the present study provided some of the first experimental evidence of the content and consequences of gender stereotypes for young children. Specifically, we found that adults rated 3- to 4-year-old children who violated gender stereotypes as less likeable than their stereotype-conforming peers. Across a host of measures, we also found that boys, in general, were rated less positively than girls, and that this effect was most pronounced for stereotype-violating boys. These results suggest that gender backlash is not limited to adults, and instead, reinforces traditional gender roles by penalizing the stereotype violations of even very young children.

## Supporting information

S1 Text Supplemental online materials. (DOCX)
