# Supplementary material for: Backlash against gender stereotype-violating preschool children
Source: PLoS One. 2018 Apr 9;13(4):e0195503. doi: 10.1371/journal.pone.0195503 (PMC5890994; doi:10.1371/journal.pone.0195503)
Supplement: S1 Text — (DOCX) [file pone.0195503.s001.docx]

Table of Contents

Supplemental Methods

Adjective for Exp. 1 Norming Study

Full materials for Exps. 2 and 3

Supplemental Results

Exp. 1: Table of gender-neutral characteristics

Exp. 2: Gender, Vignette, and Pairwise Analyses

Exp. 2: Three-way interactions

Exp. 3: Gender, Vignette, and Pairwise Analyses

**Supplemental Methods**

1. **Adjectives for Exp. 1 Norming Study**

The following adjectives were tested in the Norming study. These adjectives were organized according to our intuitions, at the outset, about which traits were likely to be pre- and pro-scriptive for girls and boys; while our intuitions were not always correct, we preserve this organization below in order to highlight (a) our strategy during stimulus design and (b) make explicit the ways in which our intuitions did (and did not) match the data.

| Girl prescriptions(required/positive feminine traits: communality) | Girl proscriptions(forbidden/negative feminine traits: dominance) | Boy  prescriptions(required/positive masculine traits: agency) | Boy  proscriptions(forbidden/negative masculine traits: weakness) |
| --- | --- | --- | --- |
| Adorable | Aggressive | Adventurous | Afraid of the dark |
| Affectionate | Argues with parents | Big appetite | Anxious |
| Caring | Bossy | Brave | Clingy with mom |
| Clean | Bratty | Clever | Cries often |
| Comforts other children when they are crying | Bruises on knees | Confident | Easily frightened |
| Enjoys cooking | Challenges authority | Determined | Emotional |
| Gentle | Dirty | Energetic | Enjoys wearing skirts and dresses |
| Good at sharing | Disobedient | Fearless | Fragile |
| Graceful | Doesn’t wait her/his turn | Handsome | Gets pushed around by other kids |
| Has good manners | Interrupts others | Independent | Has security blanket |
| Helps mom bake | Loud | Intelligent | Introverted |
| Helps out around the house | Messy | Is a leader | Is frequently sick |
| Is an “easy baby” | Plays with trucks | Likes playing outside | Likes Princesses |
| Kind | Pulls others’ hair | Likes superheroes | Likes to be held |
| Obedient | Refuses to pick up toys | Likes to play with tools | Likes to play with dolls |
| Patient | Rowdy | Likes to pretend to be a soldier | Likes to wear nail polish |
| Pays attention to what other people are wearing | Slobbers | Loves sports | Loves pink |
| Pretty | Smelly | Loves to get dirty | Needs lots of attention from adults |
| Snuggly | Sometimes hits others | Self-reliant | Picky eater |
| Sweet | Steals toys | Strong | Sensitive |
| Waits her/his turn | Stubborn | Tough | Submissive |
| Well-behaved | Thinks it’s funny when other children are crying | Unemotional | Weak |
| Frequently has a runny nose | Unbrushed hair |  | Wears tutus |
|  | Wears clothes that don’t match |  |  |

2. **Full-text of introduction and comprehension questions for Exps. 2 and 3**

“We are interested in how young children are selected for admissions to daycare and preschool programs. Children’s early education and socialization is important for their development. However, there are often more applicants than spots at preschool and daycare programs around the country. This means that preschool and daycare teachers/directors must often select which children to admit. Little is known about how people weigh various factors and make the complicated decision to accept a new students to their preschool/daycare.”

Question: Which of the following best summarizes the information above?

A. The researchers want to understand how people weigh various factors when deciding which children to admit to the daycares and preschools.

B. There are always enough slots to allow every child to attend daycare/preschool.

“To study this question, we have compiled and summarized information from **actual applications of children who have recently applied to a daycare/preschool with limited space available.** The families of these children have volunteered to share this information. We have summarized their information by creating profiles for each applicant using a standardized form, in order to adjust for differences in application procedures across daycares/preschools. Children have been assigned new names, consistent with their gender, to protect their identities. All other information (gender, age, reports about the child) is otherwise as initially reported on the preschool application”

Question: Which of the following best summarizes the information above?

A. The researchers have summarized information about the actual applications of children who have recently applied to daycares/preschools.

B. The researchers have summarized information about high school students’ test scores.

“Today, we will be assigning you to read the applicant profile of one randomly selected child from the nationwide database. Please imagine that you are actually evaluating the child’s application to join a daycare/preschool that you are in charge of. After reading the child’s profile, you will be asked to provide your opinions of the child, and offer the family feedback about moving forward with their child’s education.”

Question: Which of the following best summarizes the information above?

A. My job today is to skim a series of profiles and think about their contents.

B. My job today is to read the applicant profile of a single child, to evaluate the child’s application to join a daycare/preschool, and to provide opinions and feedback to the child.

“Remember, the national database we have created contains information both from very qualified children, as well as children who are less competitive. Thus, we ask that you be prepared to offer honest feedback to the child you are selected to read about, regardless of how qualified they are. Please do not hold back, and don’t be afraid to be truthful -- the success of the current project depends upon your ability to provide honest, straightforward feedback.”

Question: Which of the following best summarizes the information above?

A. It is best if I withhold my honest evaluation of the child.

B. Some children in the database are qualified for preschools while others may not be, and my honest and truthful feedback will ensure the success of this project.

3. **Profile Evaluations.** Participants are given 3-5 statements for each measure and asked to rate each statement on either the scale (scale: 1-7; 1 = Not at all, 7 = very much), or the scale (scale: 1-7; 1 = Not at all likely, 7 = Very much likely). Participants are instructed to “Please respond to the following statements”. Profile evaluations are participants’ evaluations of a child. All measure are self-report.

***Competence measure (cite).*** The competence scale is comprised of five statements including: ‘Did the child strike you as competent?’, ‘How likely is it that the child has the necessary skills to succeed in daycare/preschool?’, ‘How qualified do you think this child is to join a selective daycare/preschool?’, ‘To what extend do you think the child would do well in this school?’, ‘how much do you think the child would thrive in this classroom?’.

***Hireability measure (cite).*** The hireability scale is comprised of three statements including: ‘If you were running a daycare, how likely would you be to invite this child to come and interview for a spot?’, ‘If you were running a daycare, how likely would you be to give the last remaining slot to this child?’, ‘How likely do you think it is that the child was actually accepted into the daycare to which he/she applied?’.

***Likeability measure (cite).*** The likeability scale is comprised of four statements including: ‘How much do you like the child?’, ‘To what extend do you think that other children would enjoy playing with this child?’, ‘How much do you think this child’s teachers would like this child?’, ‘Would the child fit in well with other similarly aged children?’.

***Scholarship conferral.*** Participants saw: “On average, daycare costs $1000/month in this child’s region. If you had the option of giving this applicant a scholarship to attend daycare/preschool, how much of a scholarship (monthly discount on tuition) would you give?”. Participants were asked to respond by selecting a dollar amount (sliding scale: 1-1000; 0 = no scholarship, 1000 = full scholarship).

***Willingness to Interact with Child measure.*** Comprised of three statements including: ‘If you had a three year old child, how likely would you be to invite John/Jennifer over for a playdate?’, ‘How much would you be willing to babysit John/Jennifer if his/her parents asked you to?’, ‘To what extent do you think you would be annoyed by John/Jennifer?’.

***Moral Outrage measure.*** Comprised of three statements including: ‘to what extent would you describe John/Jennifer’s behavior as morally wrong?’, ‘To what extent would you describe John/jennifer’s behavior as disgusting?’, ‘To what extent were you offended by John/Jennifer’s behavior?’

***Perceptions of Parent.*** Comprised of seven statements including: ‘To what extent did you think that John/Jennifer’s parents were doing a good job raising him/her?’, ‘How much would you want to get to know John/Jennifer’s parents?’, ‘To what extent do you think that John/Jennifer’s parents are competent?’, ‘How much do you think that John/Jennifer’s parents have the skills necessary to succeed in parenting?’, ‘How qualified do you think that John/Jennifer’s parents are to be parents?’, ‘If you had to guess, to what extent do you think that John/Jennifer’s parents are too permissive?’, ‘To what extent do you think that John/Jennifer’s parents should take more control of their son/daughter’s behavior?’

**Self Evaluations.** Participants are given 3-5 statements for each measure and asked to rate each statement on either the scale (scale: 1-7; 1 = Not at all, 7 = very much), or the scale (scale: 1-7; 1 = Not at all likely, 7 = Very much likely). Participants are instructed to “Please respond to the following statements”. Profile evaluations are participants’ evaluations of themselves. All measures are self-report.

***Modern Sexism Scale.*** Comprised of 7 statements including: ‘Discrimination against women is no longer a problem in the United States.’, ‘Women often miss out on good jobs due to sexual discrimination’, ‘It is rare to see a women treated in a sexist manner on television.’, ‘On average, people in our society treat husbands and wives equally.’, ‘Society has reached the point where women and men have equal opportunities for achievement.’, ‘It is easy to understand why women’s groups are still concerned about societal limitations of women’s opportunities.’, ‘Over the past few years, the government and news media have been showing more concern about the treatment of women than is warranted by women’s actual experiences.’

***Experience with Infants and Kids scale.*** Participants were asked to rate “How much experience you had with INFANTS (18 months or under) in the last 5 years?” and “How much experience have you had with CHILDREN (18 months to 5 years) in the last 5 years?” on a scale. [scale: 0-10; 0 = no contact, 2 = seldom having been with, 9 = having had extensive contact for at least a year AND being partially responsible for their care (e.g., as with a younger sibling), 10 = having had extensive contact with and extensive responsibility for their care (e.g., raising own child, working in a daycare center)].

***Demographic Questionnaire.*** Participants were asked to report their Gender [Male/Female/Other], Parenting Status [Parent/Never Parent], Race [Black/Asian/AmericanIndian/AlaskaNative/Hawaiian/NativePacificIslander/White/TwoOrMoreRaces/PreferNotToAnswer], Political Orienation [What is your political orientation on a scale from 1 = extremely liberal to 7 = extremely conservative], English Fluency [Is your English your first language Yes/No], Country of Origin [what is your country of origin/where were you born: US or if ‘Other’ Please Specify]. Say why we included each question in this questionnaire.

***Follow-up Questionnaire.*** Included to prove for suspicions regarding the purpose of the study. Any participants whose response suggested awareness of the purpose of the task or purpose of the study were excluded.

**Supplemental Results**

**Experiment 1: Gender-neutral characteristics**

**
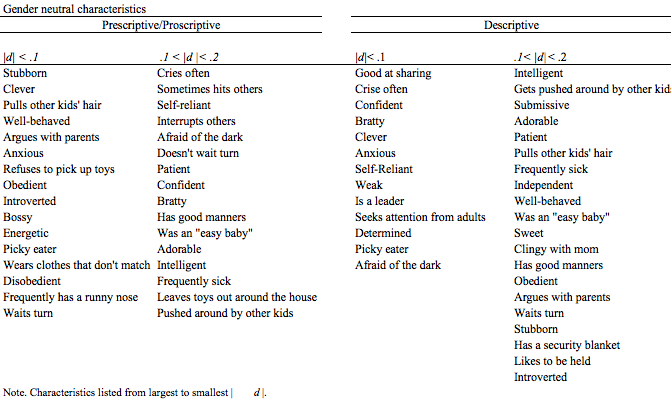
**

**Experiment 2: Gender, Vignette, and Pairwise Effects**

*Likeability.* Our ANOVA revealed a significant effect of gender, (*F*(1, 591) = 9.29, *p* = .002). Providing strong evidence of backlash, using Tukey’s HSD we found that feminine girls were rated as significantly more likeable (*M*_GirlUnmas_ = 6.02) than stereotype-violating masculine girls (*M*_GirlMasc_ = 5.50, *p* = .0003). Of interest, consistent with prior research revealing the “women are wonderful” effect (i.e. that stereotype-conforming adult women are generally liked more than adult men; Eagly & Mladinic, 1994), Feminine girls were rated as more likeable than both types of boys as well ( *M*_BoyFem_ = 5.29, *p* < .0001; *M*_BoyMasc_ = 5.69, *p* = .04). Also providing evidence of backlash, we found that stereotype-conforming Masculine boys were rated as significantly more likeable than stereotype-violating Feminine boys (*p* = .009).

*Hireability.* Consistent with the Women are Wonderful effect, Tukey’s HSD revealed that Feminine girls (*M*_GirlUnmas_ = 5.69) were rated as significantly more hireable than Masculine boys (*M*_BoyMasc_ = 5.22, *p* = .008). Demographic factors did not impact these findings.

*Willingness to Interact.* Here, we found a significant effect of Vignette (*F*(1,591) = 14.32, *p* = .0002). Tukey’s HSD revealed that participants were significantly more willing to interact with Feminine girls (*M*_GirlUnmas_ = 5.72) than Masculine boys (*M*_BoyMasc_ = 5.15, *p* = .0005) and than Masculine girls (*M*_GirlMasc_ = 5.20, *p* = .002).

**Experiment 2: Three-way interactions**


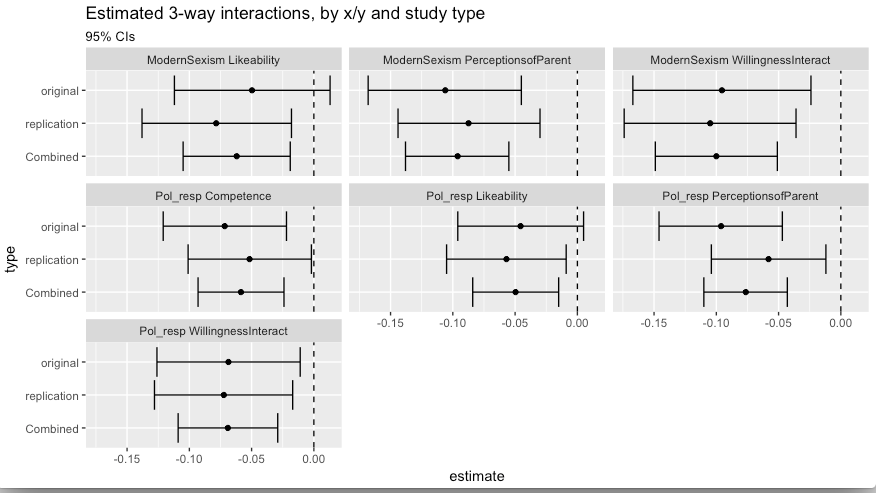


**Experiment 3: Gender, Vignette, and Pairwise Effects**

*Likeability.* An ANOVA revealed an effect of gender (*F*(1,615) = 13.73, *p* = .0002, CI: ,η^2^ = .02). As in Experiment 2, we found that feminine girls were rated as significantly more likeable (*M*_GirlFem_ = 5.9) than feminine boys (*M*_BoyFem_ = 5.4; *p* = .0004; *d* = .46). New to this experiment, we also found that stereotype-violating masculine girls (*M*_GirlMasc_ = 5.7) were rated as significantly more likeable than feminine boys (*p =* .03; *d* = .32); there were no other significant pairwise effects. There were no significant three-way interactions of participant demographics, vignette, and applicant gender.

*Willingness to Interact*. While there was no evidence of backlash effects, we found evidence of an effect of vignette on Willingness to Interact (*F*(1,614) = 5.73, *p* = .017, η^2^= .009), and an effect of Gender Willingness to Interact (*F*(1,614) = 7.06, *p* = .008, CI: , η^2^= .011).

*Competence.* New to this experiment, we found significant unexpected effects of gender on Competence (*F*(1,616) = 11.56, *p* = .0007, η^2^=.018), Hireability (*F*(1,614) = 9.46, *p* = .002, CI: , η^2^= .015).

*Perceptions of Parents*. We found effects of gender and Perceptions of Parents (*F*(1,614) = 4.85 *p* = .028, CI: , η^2^=.008 ), such that girls received consistently higher scores than boys.

*Hireability, Moral Outrage, Scholarship Conferral.* As in Experiment 2, we found no evidence of backlash or of effects of gender/vignette against children for any of our other DVs (hireability, moral outrage).
